# Preparation of Cross-Sectional Membrane Samples for Scanning Electron Microscopy Characterizations Using a New Frozen Section Technique

**DOI:** 10.3390/membranes13070634

**Published:** 2023-06-29

**Authors:** Hongyun Ren, Xian Zhang, Yi Li, Dandan Zhang, Fuyi Huang, Zixing Zhang

**Affiliations:** 1 Center of Analytical Instrument, Institute of Urban Environment, Chinese Academy of Sciences, Xiamen 361021, China; hyren@iue.ac.cn (H.R.); xzhang@iue.ac.cn (X.Z.); ddzhang@iue.ac.cn (D.Z.); fyhuang@iue.ac.cn (F.H.); 2 Key Lab of Urban Environment and Health, Institute of Urban Environment, Chinese Academy of Sciences, Xiamen 361021, China; 3 School of Chemical Engineering and Technology, Sun Yat-sen University, Zhuhai 519082, China; liyi266@mail.sysu.edu.cn

**Keywords:** polymeric membrane, scanning electron microscopy, preparation of membrane cross-sections, frozen section technique, embedding mold, embedding medium

## Abstract

Characterization of the cross-sectional morphologies of polymeric membranes are critical in understanding the relationship of structure and membrane separation performances. However, preparation of cross-sectional samples with flat surfaces for scanning electron microscopy (SEM) characterizations is challenging due to the toughness of the non-woven fabric support. In this work, a new frozen section technique was developed to prepare the cross-sectional membrane samples. A special mold was self-designed to embed membranes orientationally. The frozen section parameters, including the embedding medium, cryostat working temperature, and sectioning thickness were optimized. The SEM characterizations demonstrated that the frozen section technique, using ultrapure water as the embedding medium at a working temperature of −30 °C and a sectioning thickness of 0.5 µm, was efficient for the preparation of the membrane samples. Three methods of preparation for the cross-sectional polymeric membranes, including the conventional liquid nitrogen cryogenic fracture, the broad ion beam (BIB) polishing, and the frozen section technique were compared, which showed that the modified frozen section method was efficient and low cost. This developed method could not only accelerate the development of membrane technology but also has great potential for applications in preparation of other solid samples.

## 1. Introduction

Polymeric membranes are widely used for the separation and purification of solutes in a solution or for the separation of colloidal suspensions [1]. Membrane separation technology has become an overwhelming technology owing to its advantages of being a simple process, no pollution, low energy consumption, and high efficiency [2]. Their applications cover the chemical industry, food processing, water treatment, biomedicine, and so on [3]. Based on the particle size that is intercepted in raw water, membranes can be subdivided into microfiltration (MF), ultrafiltration (UF), nanofiltration (NF), and reverse osmosis (RO) membranes [4]. Polymeric membranes generally consist of two structural layers. A functional layer mostly synthesized from organic polymers (such as polypropylene, polyvinylidene fluoride, polysulfone, polyethersulfone, and polyamide), which has dense micropores and the ability of intercepting particles or macromolecules. A support layer with a large through-holes structure is usually created with polyethylene (PE) non-wovens, which can improve the strength of the membranes [5,6,7]. In practical applications, it is necessary to develop membranes with different functions by modifying their structures, which include the porosity, pore size, modified materials, interfacial polymerization coatings on the membrane surface, etc. These are the key factors that influence the performance of membranes in terms of permeability, separation effect, hydrophilicity, and antifouling [8,9]. A lot of structural information can be obtained by characterizing the membrane cross-sections [10,11,12]. The scanning electron microscope (SEM) is an effective technical method to characterize the morphology of membranes [7,13,14]. Therefore, it is significant to prepare SEM cross-sectional samples with the original structure of the membrane.

At present, some methods were reported to prepare membrane cross-sections. The most commonly used method is cryogenic fracture after immersion in liquid nitrogen for a few minutes. The fracture surfaces prepared by this method are free of stress damage; moreover, this method is fast and easy to operate [15,16,17]. However, the cross-sections of the membranes that break with this method are uneven and random—there may be debris left or the membrane may bend. In particular, membranes with a tough non-woven support layer require a greater force to be broken, resulting in a scattered distribution of broken fibers. These shortcomings can lead to an inaccurate morphological characterization using SEM and a composition analysis using energy dispersive spectroscopy (EDS). Another method for preparing cross-sectional samples is the broad ion beam (BIB) polishing, based on the principle of ion bombardment [18]. It has the advantages of low strain, low contamination, and accurate positioning. This method was used to polish membranes at low temperatures with an argon ion source to obtain cross-sectional samples [19,20,21,22]. BIB polishing processes areas with a depth of about 100 µm and a width of 3–4 mm taking several hours or more [19,23]. Moreover, this technique has the characteristics of a small processing range, long processing time, and high cost. Therefore, this method is not yet widely applied in the preparation of cross-sectional membrane samples. How to improve the quality of the cross-sections of the prepared membranes and to reduce the time, as well as the cost of sample preparation are still challenging.

Here, we developed a modified frozen section technique for the preparation of cross-sections of polymeric membranes. This technique was based on a self-designed mold and a cryotome. The mold was fabricated to ensure that the membrane could be cut vertically. The embedded blocks of the membranes were sectioned at low temperature using a cryotome to remove the deformed part of the cross-sectional surface. Since the SEM and EDS analysis require the sample to be free of other interfering substances, the embedding medium used in this method must be one that can be removed without affecting the sample structure. Therefore, traditional embedding mediums, such as gelatin [24] and OCT (a water-soluble mixture of polyethylene glycol and polyvinyl alcohol) [25] need to be replaced by a new one. Sucrose solution is a common embedding medium for samples during a cryogenic transmission electron microscopy analysis [26] and can be diluted with water and washed off. The sucrose solution also acted as a cryoprotectant to prevent the formation of ice crystals during freezing to avoid a secondary porosity on the sample [26]. Consequently, several concentrations of sucrose solutions were investigated to determine the optimal concentration. In addition, different temperatures and the thicknesses of fine cutting were explored. The cross-sections of the MF, UF, and RO membranes prepared by the three different techniques (conventional liquid nitrogen cryogenic fracture method, BIB polishing, and the modified frozen section technique) were compared. The aim of this study is to develop an efficient method for the preparation of membrane cross-sections to contribute to the evolving research on membranes. This technique can also be applicable to other solid samples, providing a low cost tool for cross-sectional sample preparation for SEM characterizations.

## 2. Materials and Methods

### 2.1. Materials

Three kinds of membranes were used in this study, namely MF, UF, and RO membranes. The MF and UF membranes used in this experiment were kindly provided by Kai-Song Zhang’s group at the Institute of Urban Environment, Chinese Academy of Sciences. The commercial RO membrane was obtained from SunRain Group Ltd. The MF membrane consists of a polyvinylidene fluoride (PVDF) functional layer and a PE non-woven support layer. The pore sizes of the MF membrane ranges from 0.1 μm to 10 µm. The UF membrane is composed of a polyethersulfone (PES) functional layer and a PE non-woven support layer with its pore sizes ranging from 2 nm to 100 nm. The RO membrane consists of a polyamide (PA) functional layer, a porous polysulfone (PSf) sublayer, and a PE non-woven support layer, and its pore sizes range from 0.1 nm to 1 nm. The sucrose used was of analytical grade, provided by Sinopharm Chemical Reagent Co., Ltd. All the ultrapure water used in this research was prepared by the ultrapure water system (A10, Milli-Q, Lyon, France).

### 2.2. Liquid Nitrogen Cryogenic Fracture

The membranes were cut into narrow strips of about 20 mm × 7 mm with scissors, with a slit cut in the middle of each strip. The strips were frozen in liquid nitrogen for 5 min, then taken out and pulled from both ends with pliers until the membrane broke in two.

### 2.3. BIB Polishing

The three types of membranes were polished with the Ar^+^ ion beam at a low temperature using a BIB milling (IM4000Plus, Hitachi, Tokyo, Japan). Membranes were cut into rectangles of about 5 mm × 7 mm with scissors and were wrapped in several layers of aluminum foil. The prepared membrane samples were fixed between the sample holder and the shield plate. The setting parameters of the BIB milling for processing the MF and RO membranes: temperature −40 °C and voltage 4 kV, 3 h. The setting parameters for the UF membrane: temperature −40 °C and voltage 4 kV, 5 h.

### 2.4. Frozen Section Technique

#### 2.4.1. Fabrication of the Embedding Mold

**Figure 1:**
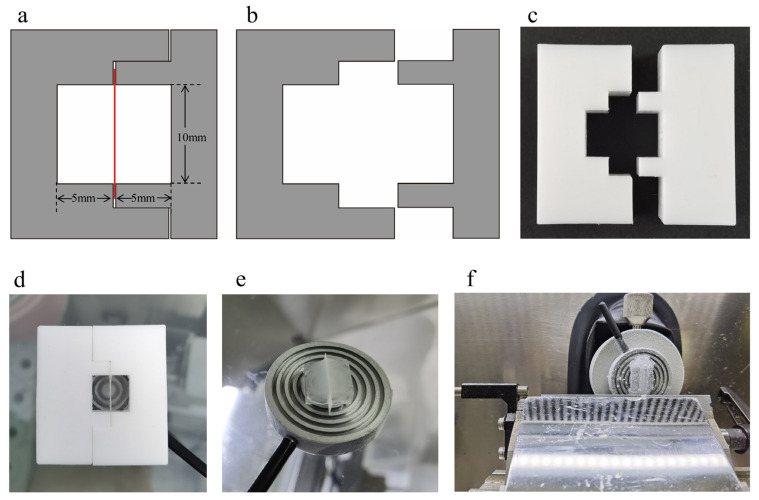
The structure of the embedding mold (**a**,**b**). (**a**) The top view of the embedding mold. The red part indicates the membrane. (**b**) The top view of the two separate parts of the mold. (**c**) The physical object of the embedding mold made of silicone rubber. (**d**) The membrane was fixed vertically by the embedding mold and frozen into an embedding block together with the embedding medium. (**e**) Embedding block after removing the embedding mold. (**f**) The trimmed embedding block was mounted on the cold head.

An embedding mold, which can fix the membrane vertically and load the embedding medium on the specimen holder, was designed in this work to obtain a perfect vertical cross-section of the membranes. The mold consists of a mortise and tenon structure (Figure 1a–c), which can be assembled and disassembled easily. It is suitable for holding samples with different thicknesses and can be customized according to the sample size. The mold is made of silicone rubber, which can resistant low temperatures. A mold that could obtain an embedding block of 10 mm × 10 mm × 10 mm was fabricated as shown in Figure 1.

#### 2.4.2. Use of the Embedding Mold and Preparation of Membrane Cross-Sections

Sucrose solutions of 10%, 20% and 30% were prepared using mass ratio in ultrapure water. The sucrose solutions and the ultrapure water were used as embedding mediums, respectively. The cryostat microtome (SYD-K4080, Shenyang Yude, Shenyang, China) was used to cut the membranes to obtain the cross-sections. First, the samples were cut into rectangles of approximately 16 mm × 6 mm and immersed in the embedding medium for 30 min. The cryotome was pre-cooled until the temperature of the sample chamber, the cold head on the robot arm, the cryo-stage and microtome blade reached the desired value. Next, the membrane was fixed vertically on the sample holder in the chamber of the cryotome with the mold, followed by the addition of the embedding medium. Due to the low temperature of the sample holder (−30 °C), the embedding medium froze immediately when it was added to the mold without flowing out of the gap between the mold and the sample holder (Figure 1d). After the embedding medium was frozen, the mold was removed (Figure 1e). Then the ends of the membrane that were none immersed in the embedding medium were cut off, followed by adding the embedding medium to the ends using a pipette so that the exposed ends were also covered. The excess embedding block around the membrane was trimmed to make it pyramidal for sectioning (Figure 1f). After that, the sample holder was mounted on the cold head with the membrane perpendicular to the direction of the blade. The embedding block was trimmed to fully expose the entire cross-section. The sample was moved to the unused cutting edge, and the section thickness was set to the desired value, with the sample cut twice. The remaining membrane on the sample holder was the target samples instead of the cut sections. After thawing the embedding medium, the membranes were rinsed three times with ultrapure water (5 min for each rinse). Finally, the samples were dried at 35 °C.

### 2.5. SEM Observation and EDS Analysis

Field emission scanning electron microscopy (SEM, S−4800, Hitachi, Tokyo, Japan) was used to investigate the morphologies of the membrane cross-sections that were prepared using the different methods. Before the SEM observation, the dry membrane cross-sections were sputtered with gold to increase conductivity. The elemental mapping of the membrane cross-sections was studied using energy dispersive X-ray spectroscopy (EDS, EX250, Horiba, Tokyo, Japan).

## 3. Results and Discussion

### 3.1. Cross-Sections of Membranes Cryogenically Fractured with Liquid Nitrogen

**Figure 2:**
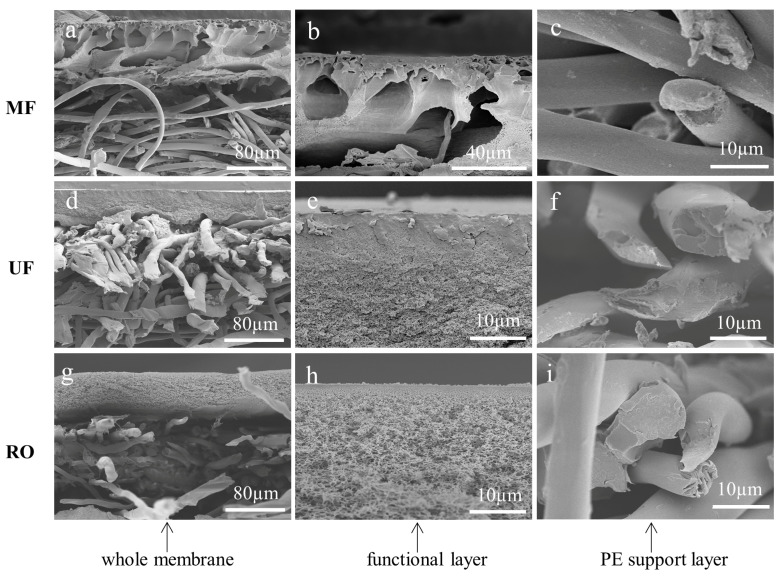
SEM images of the cross-sections of MF membrane (**a**–**c**), UF membrane (**d**–**f**), and RO membrane (**g**–**i**) prepared by the conventional liquid nitrogen cryogenic fracture method. (**b**,**c**) The PVDF functional layer and the PE support layer of MF membrane, respectively. (**e**,**f**) The PES functional layer and the PE support layer of UF membrane. (**h**,**i**) The PA functional layer, porous PSf sublayer and the PE support layer of RO membrane.

The cross-sections of MF, UF, and RO membranes were prepared by the most commonly used method: the liquid nitrogen cryogenic fracture. Figure 2 shows the cross-sectional morphologies of the membranes. A neat and flat cross-section was desired to accurately analyze the thickness and the pore structure of the polymeric membranes. It could be found that the functional layer and the support non-woven fabric were disordered for all the membranes cryogenically fractured with liquid nitrogen as the PE non-woven fabric was too tough to be broken. As shown in Figure 2a, the PVDF functional layer of the MF membrane had an asymmetric structure with macropores across the section. However, the details of the top dense layer were not clear as the PVDF crust layer was partially covered on the top surface (Figure 2b), which was caused by the fracture force. Moreover, the cross-section of the non-woven fabric and the PVDF functional layer was not in coplanar (Figure 2a–c). These features result in the size, shape, and distribution of the pores as well as the thickness of the active layer could not be accurately determined. Figure 2d shows that the UF membrane with a dense PES active layer was supported by a PE support. Nevertheless, neither the dense PES active layer nor the fabric support was perfectly fractured. There were cracks and debris on the cross-section of the PES layer (Figure 2e), while the non-woven fabric was deformed (Figure 2f). The same phenomenon has also been found for the RO membrane (Figure 2g), which consists of a nanoscale thin film layer, a porous PSf sublayer, and a PE support layer. The top polymeric PSf layer separated from the fabric support, leading to the observation of the overall structure of the thin film composite RO membrane challenging. In addition, the cross-sectional SEM image of the PSf layer was scattered and severely affected the analysis of the real pore structure and sizes of the sublayer (Figure 2h). The penetration of the PSf into the porous non-woven support couldn’t be observed (Figure 2i). Overall, the conventional liquid nitrogen cryogenic fracture method is not favorable for sample preparations for tough materials.

### 3.2. Cross-Sections of Membranes Prepared by BIB Polishing

**Figure 3:**
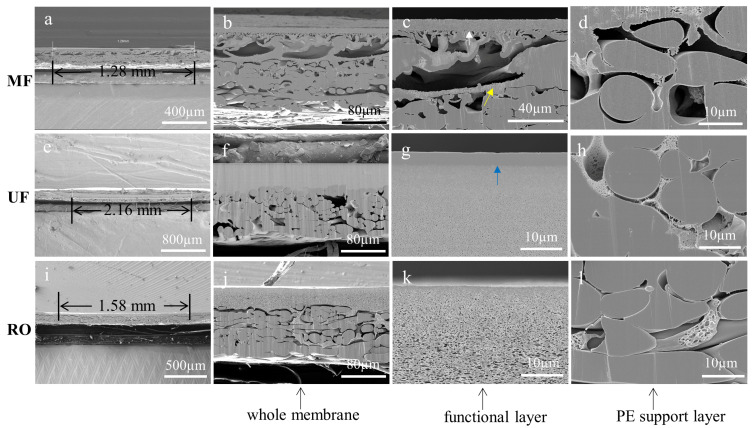
Cross-sectional SEM images of the MF, UF, and RO membranes. The effective cross-sectional area (**a**,**e**,**i**) and the results of the cross-sections of the membranes processed by BIB milling (**b**–**d**,**f**–**h**,**j**–**l**). The white arrow indicates the residue of the sample preparation. The blue arrow indicates the skin layer of the UF membrane. The yellow arrow indicates the trace damages.

As a cutting method that minimizes mechanical damage, BIB polishing can be used to prepare the cross-sections of MF, UF, and RO membranes. In this work, their effective processing widths were 1.28 mm, 2.16 mm, and 1.58 mm, respectively, and the effective depths were the thickness of the membranes as shown in Figure 3a,e,i. The white arrow shown in Figure 3c was the residue of the sample preparation. It could be found that all the membranes had well-cut cross-sections of the sublayer (Figure 3c,g,k) and the non-woven fabric (Figure 3d,h,l). Different from the liquid nitrogen cryogenic fracture method, the non-woven fabric prepared by BIB polishing had a flat cross-section, which could reflect the intrinsic structure without damaging the top functional layer. Furthermore, the interface between the functional layer and the non-woven fabric was clear. The yellow arrow indicates the trace damages. However, the cross-sections of the UF and RO membranes were not completely flat due to the different angles of ion beam bombardment as shown in Figure 3f,j. Apart from the minor defects mentioned above, the cross-sections of the membrane prepared by BIB polishing had minimal stress-strain and were free from contamination. The BIB polishing has limitations in obtaining defect-free cross-sectional samples though it is advantageous to that of the liquid nitrogen cryogenic fracture method.

### 3.3. Cross-Sections of Membranes Prepared by Frozen Section Technique

#### 3.3.1. Effect of Embedding Medium on the Cross-Sections of Prepared Membranes

The embedding medium is crucial to the frozen section technique as it adheres well to the sample structure and provides a framework that acts as a fixation and support [27]. Chen et al. used a sucrose solution as an embedding medium, and used a cryoultramicrotome to microtome the polymer film into ultrathin sections of 50 nm thickness for the transmission electron microscopy characterization [28]. The sample had to be trimmed into blocks of a few hundred microns or even smaller to fit the sectioning range of the cryoultramicrotome [29,30,31]. However, the resultant cross-sectional area was too small for the SEM analysis. In this work, the commonly used cryotome in clinical pathology diagnosis was adopted to cut the membranes, which can have a sectioning area of 30 mm × 30 mm [32]. The microscopy of soft materials, such as hydrogels, can be affected by artefacts due to ice crystals formed during freezing [33,34]. Kaberova et al. investigated the causes of artefacts in the characterization of hydrogels using SEM and showed that hydrogels with a tensile modulus as a mechanical parameter below 0.5 MPa or equilibrium water content above 100% are prone to ice crystals due to freezing [35]. However, the tensile modulus of the polymers is mostly greater than 420 Mpa and the equilibrium moisture content is mostly less than 15% [36]. Therefore, we had included ultrapure water, which is not cryoprotective, in the range of embedding medium and dried the samples directly. The sucrose solutions with concentration of 0%, 10%, 20%, and 30% were used as the embedding medium to investigate the impact of concentration on the cross-section of the prepared membranes. The working temperature of the cryotome was set at −30 °C, and the fine cutting thickness was set to 0.5 µm.

**Figure 4:**
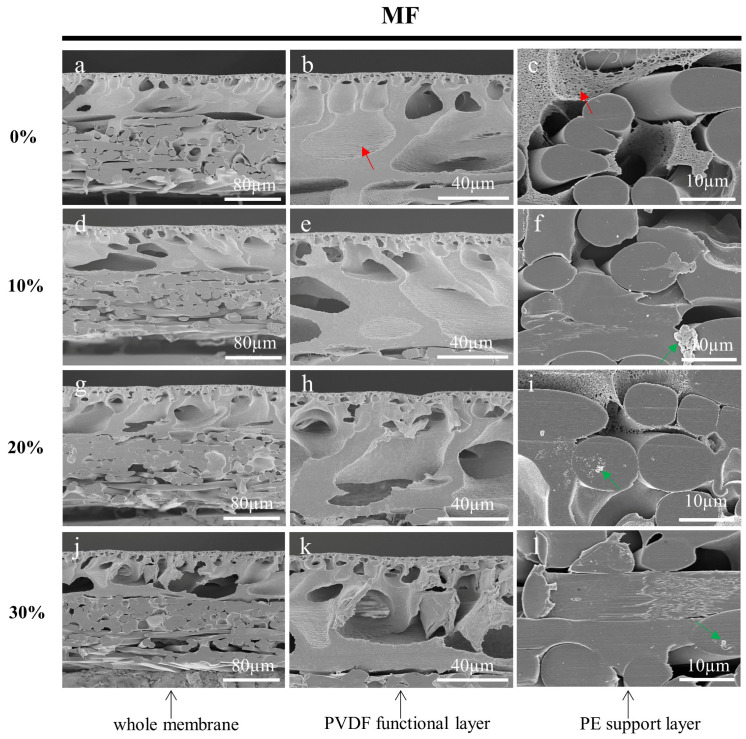
Cross-sectional SEM images of MF membranes embedded in 0% (**a**–**c**), 10% (**d**–**f**), 20% (**g**–**i**), and 30% (**j**–**l**) sucrose solutions, respectively and sectioned by a cryotome. The red arrows indicate the large pores on the membrane cross-sections. The green arrows show the impurities.

**Figure 5:**
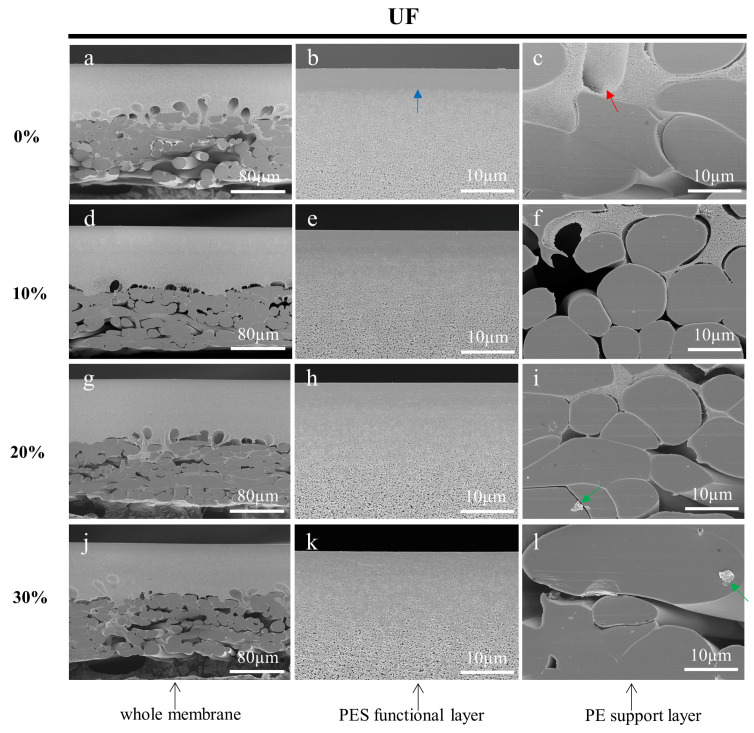
Cross-sectional SEM images of UF membranes embedded in 0% (**a**–**c**), 10% (**d**–**f**), 20% (**g**–**i**), and 30% (**j**–**l**) sucrose solutions, respectively and sectioned by a cryotome. The blue arrow indicates the skin layer of the membrane. The red arrows indicate the large pores on the membrane cross-sections. The green arrows show the impurities.

**Figure 6:**
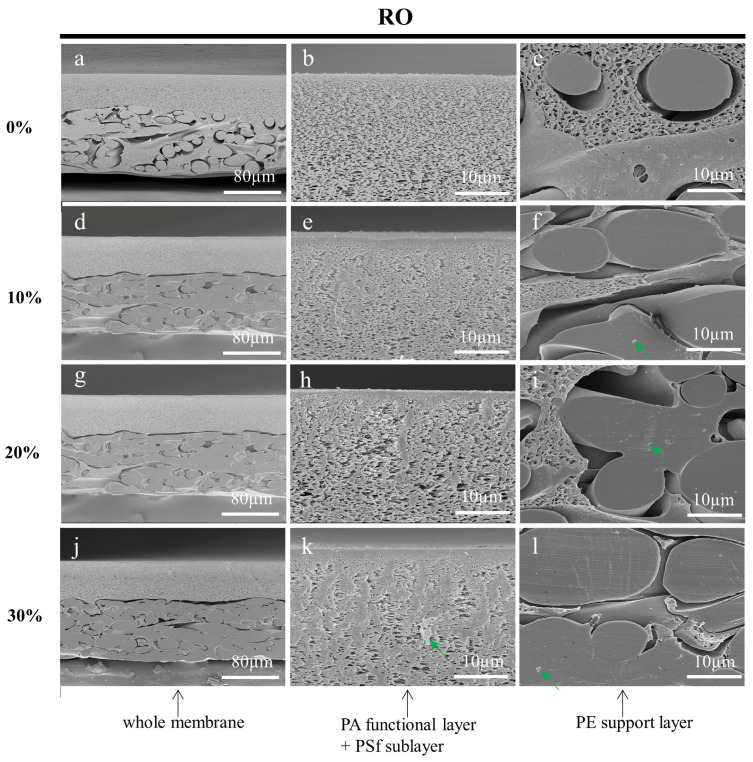
Cross-sectional SEM images of RO membranes embedded in 0% (**a**–**c**), 10% (**d**–**f**), 20% (**g**–**i**), and 30% (**j**–**l**) sucrose solutions, respectively and sectioned by a cryotome. The green arrows show the impurities.

After rinsing and drying, the prepared membranes were characterized using SEM. Figure 4, Figure 5 and Figure 6 shows the cross-sections of MF, UF, and RO membranes, respectively. As the concentration of sucrose solution increased, the stress damage in the cross-section of the three membranes became more obvious. The membrane cross-sections prepared with 10%, 20%, and 30% sucrose solutions as an embedding medium had some residual impurities as indicated by the green arrows. As shown in Figure 4, it could be clearly seen that the pores of the PVDF functional layer in the cross-sections of the MF membranes prepared with the low concentration of sucrose solution gradually became larger from top to bottom. Figure 5b,e,h,k displayed that the demarcation line of the skin layer of the UF membrane became less obvious as the concentration of the sucrose solution increased. However, the skin layer of the UF membrane (blue arrow) in Figure 5b prepared with ultrapure water as the embedding medium could be accurately measured. As shown in Figure 6, the functional layer of the RO membrane consists of loose and uniform structures, and the structures of the cross-section of the RO membrane prepared with the sucrose solution as the embedding medium were squeezed by stress and formed many stacks. In conclusion, all specimens prepared using ultrapure water as the embedding medium had less stress damage, and were free from contamination. The SEM images also showed that some polymer from the functional layer had infiltrated into the PE support layer. The inside of the large pores, indicated by the red arrows, had not been touched by the slicing knife, thus preserving the original structure and facilitating the observation and analysis of the membranes.

The factors that affect the sectioning performance of the embedding medium include the permeability of the embedding medium, the hardness, and the elasticity and cutting properties of the formed embedding block [37,38,39,40]. The higher the concentration of sucrose solution, the more difficult it was to completely infiltrate the internal pores of the membrane. In addition, the hardness of the frozen sucrose solution with a high concentration was lower than that of the frozen sucrose solution with a low concentration at the same temperature [41], and the sucrose solution can reach a hardness suitable for slicing below −60 °C [42]. Therefore, the sucrose solution could not fix and support the membrane structure well within the temperature range of this cryotome. As the cross-sectional samples of the SEM were much larger than the sections, it was difficult to wash off the sucrose solution completely and it was easy to leave contaminants on the membrane cross-sections. The ultrapure water, as the embedding medium, did not have the above problems. Furthermore, no artefacts due to ice crystals and drying were found in the cross-sections of the membranes prepared with the ultrapure water as the embedding medium. The results showed that the membrane cross-sections prepared using the ultrapure water as the embedding medium were satisfactory.

#### 3.3.2. Effect of Working Temperature on the Cross-Sections of Prepared Membranes

**Figure 7:**
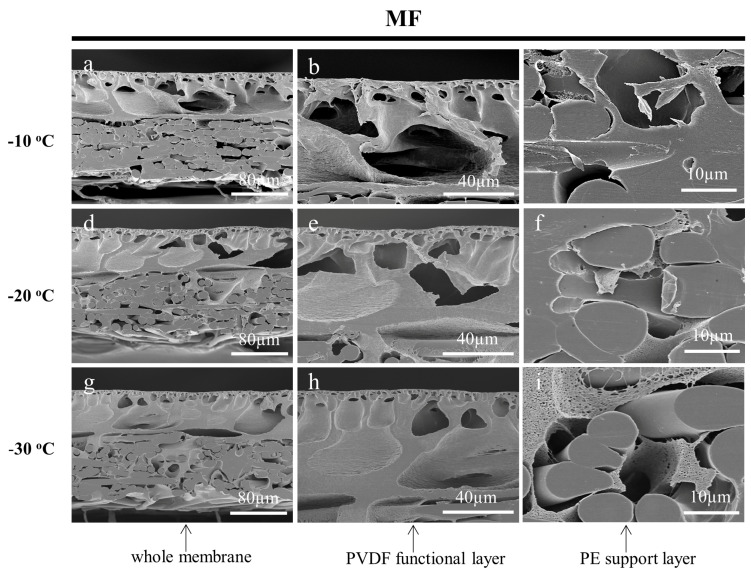
SEM images of the cross-sections of the MF membrane, which were cut by the cryotome at the temperature of −10 °C (**a**–**c**), −20 °C (**d**–**f**), and −30 °C (**g**–**i**) respectively.

**Figure 8:**
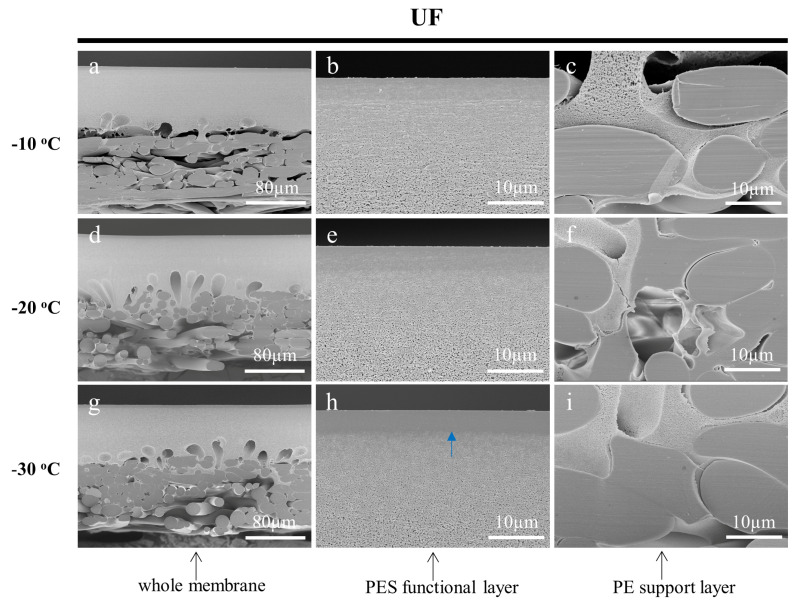
SEM images of the cross-sections of the UF membrane, which were cut by the cryotome at the temperature of −10 °C (**a**–**c**), −20 °C (**d**–**f**), and −30 °C (**g**–**i**) respectively. The blue arrow shows the skin layer of the membrane.

**Figure 9:**
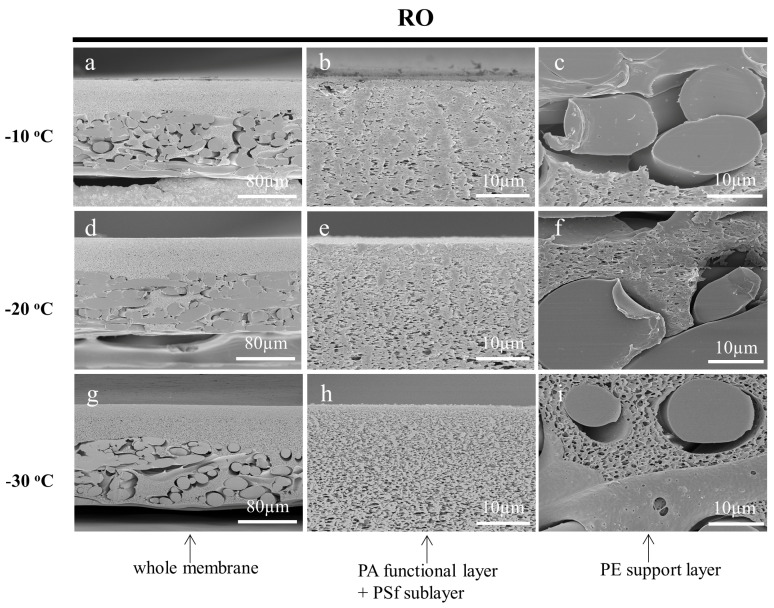
SEM images of the cross-sections of the RO membrane, which were cut by the cryotome at the temperature of −10 °C (**a**–**c**), −20 °C (**d**–**f**), and −30 °C (**g**–**i**) respectively.

One of the most important variables to obtain quality prepared samples is the temperature for the frozen section technique. Various types of samples have different cutting properties and the appropriate temperature needs to be selected according to the sample characteristics [43]. To investigate the effect of temperature on the cross-sectioning of the membrane samples, the working temperature of the cryotome was set to −10 °C, −20 °C, and −30 °C, respectively. The fine cutting thicknesses were all set to 0.5 µm and the ultrapure water was used as the embedding medium. The cross-sectional SEM images of the prepared MF, UF, and RO membranes are shown in Figure 7, Figure 8 and Figure 9, respectively. With the increase in the operating temperature, the stress deformation of all the cross-sections was enhanced. For instance, the cross-sections of the MF membrane cut at −10 °C and −20 °C were out of shape as shown in Figure 7a–f. While, the cross-section cut at −30 °C was flat and maintained its original structure without noticeable damages (Figure 7g–i). As for the UF membrane, the low working cut temperature greatly affected the observation of the crust layer. It could be interestingly found that the UF membrane cut at −30 °C had a very clear dense surface layer on the porous layer (Figure 8g–i). This result was rarely reported, as the cross-section of the UF membrane samples were generally prepared by the liquid nitrogen cryogenic fracture, which had a structure similar to the SEM images of Figure 8a–f. The cryotome at a low temperature could provide more details on the structure of the UF membranes. The cross-section of the RO membrane prepared using different temperatures also showed significant differences. It could be found that the membrane was deformed seriously at −10 °C and −20 °C as shown in Figure 9. Nevertheless, the cross-section was flat and clear when cut at −30 °C, indicating that the working temperature of the cryotome plays a crucial role in maintaining the internal structure of the polymeric samples. A low cryotome temperature of −30 °C was demonstrated to be sufficient to obtain good cross-sections for the MF, UF, and RO membranes.

#### 3.3.3. Effect of Fine Cutting Thickness on the Cross-Sections of Prepared Membranes

**Figure 10:**
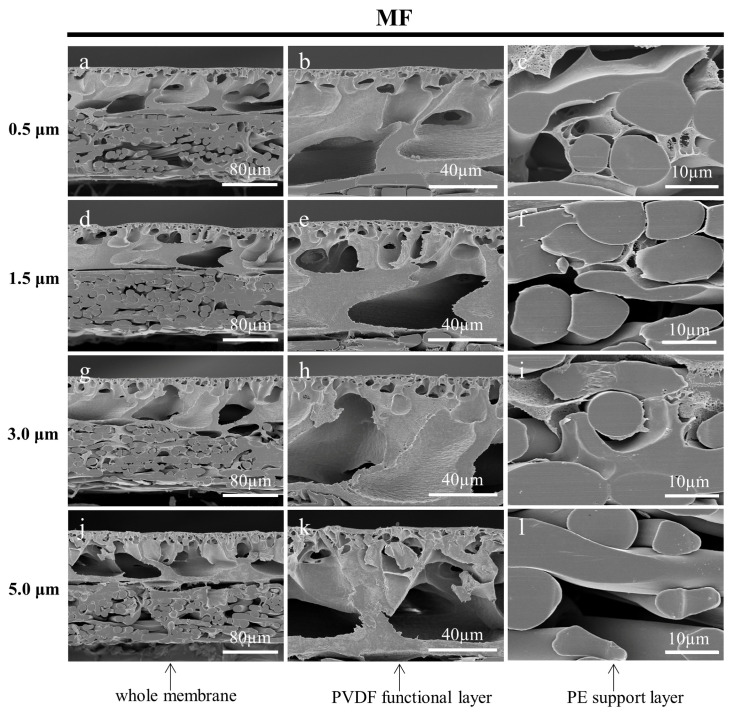
SEM images of the cross-sections of the MF membrane, which were cut by the cryotome at 0.5 µm (**a**–**c**), 1.5 µm (**d**–**f**), 3.0 µm (**g**–**i**), and 5.0 µm (**j**–**l**) thickness to remove the deformation of cross-sections, respectively.

**Figure 11:**
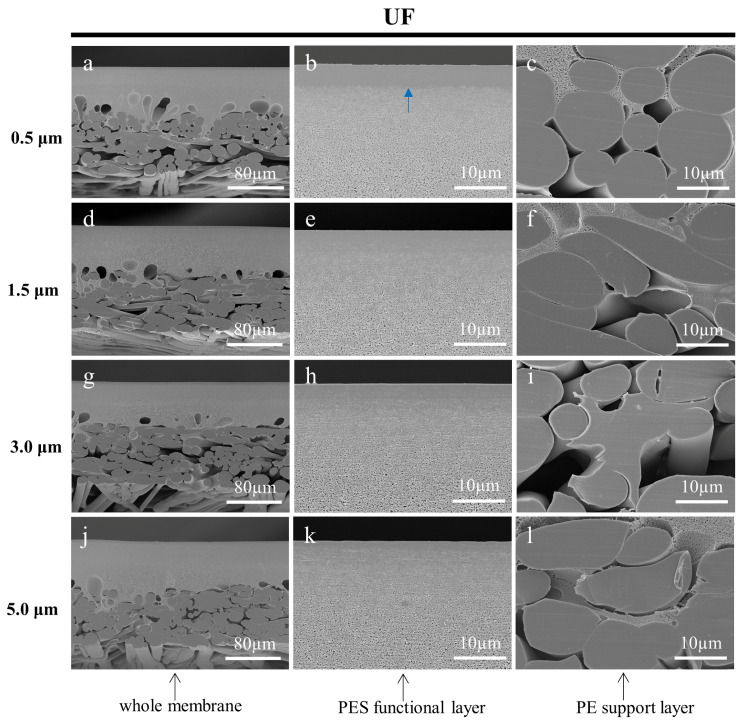
SEM images of the cross-sections of the UF membrane, which were cut by the cryotome at 0.5 µm (**a**–**c**), 1.5 µm (**d**–**f**), 3.0 µm (**g**–**i**), and 5.0 µm (**j**–**l**) thickness to remove the deformation of cross-sections, respectively. The blue arrow shows the skin layer of the membrane.

**Figure 12:**
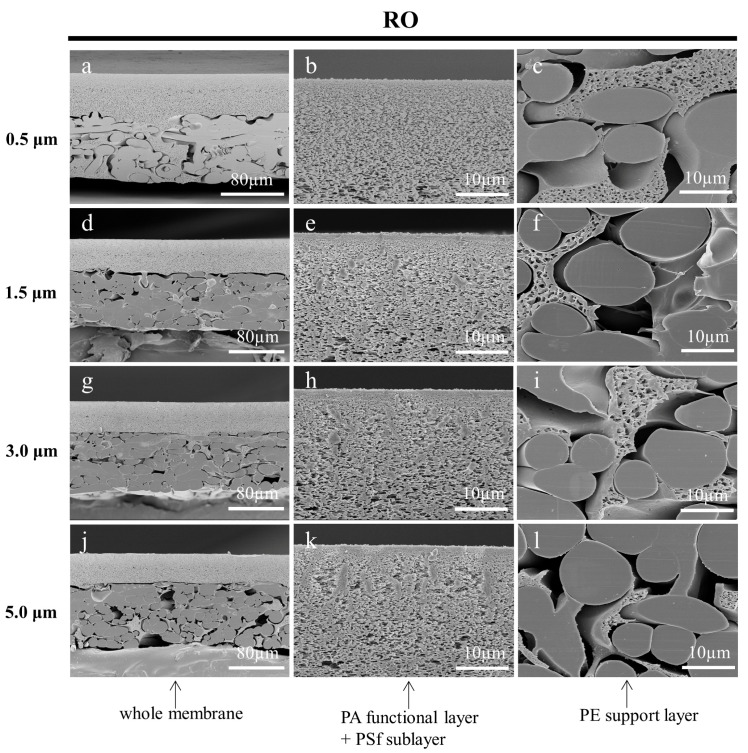
SEM images of the cross-sections of the RO membrane, which were cut by the cryotome at 0.5 µm (**a**–**c**), 1.5 µm (**d**–**f**), 3.0 µm (**g**–**i**), and 5.0 µm (**j**–**l**) thickness to remove the deformation of cross-sections, respectively.

In this work, the cryotome was innovatively used to cut off the deformed portion of the cross-section, which has a section thickness ranging from 0.5 µm to 100 µm. In the preparation of a frozen section of biological samples, the thickness of the section needs to be chosen according to the continuous section-forming nature of the sample, and the effectiveness of the tissue structure to be observed [43,44]. However, the choice of thickness at which to section the membrane samples was an issue worthy of investigation. The fine cutting thicknesses (section thicknesses) of 0.5 µm, 1.5 µm, 3.0 µm, and 5.0 µm were adopted to prepare the cross-sectional samples of the MF, UF, and RO membranes, respectively. Ultrapure water was used as the embedding medium, and the working temperature of the microtome was set to −30 °C. It could be found that the cross-sections of the membranes prepared with a smaller fine cutting thickness had more neat cuts as shown in Figure 10, Figure 11 and Figure 12. On the contrary, there were more irregular adhesion parts at the cuts, especially for the functional layers of the three membranes. The deformed part of the membrane cross-sections prepared with larger thicknesses of the fine cutting covered part of the microstructure, which affected the observation of the original structure. In addition, the skin layer of the UF membrane became increasingly inconspicuous. The result suggests that selecting a smaller fine cutting thickness can improve the quality of the membrane cross-sections prepared by the frozen section technique.

### 3.4. EDS Elemental Analysis

**Figure 13:**
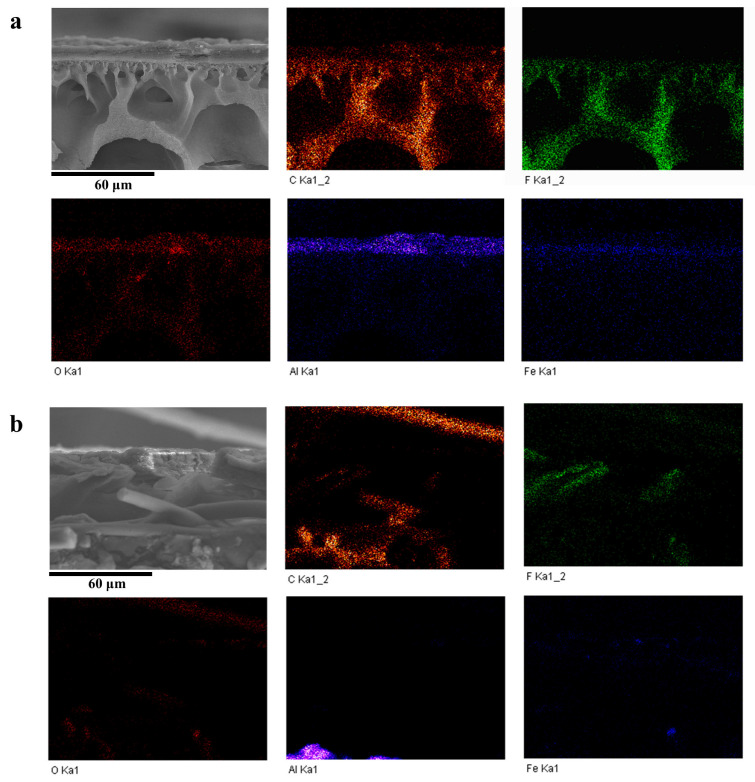

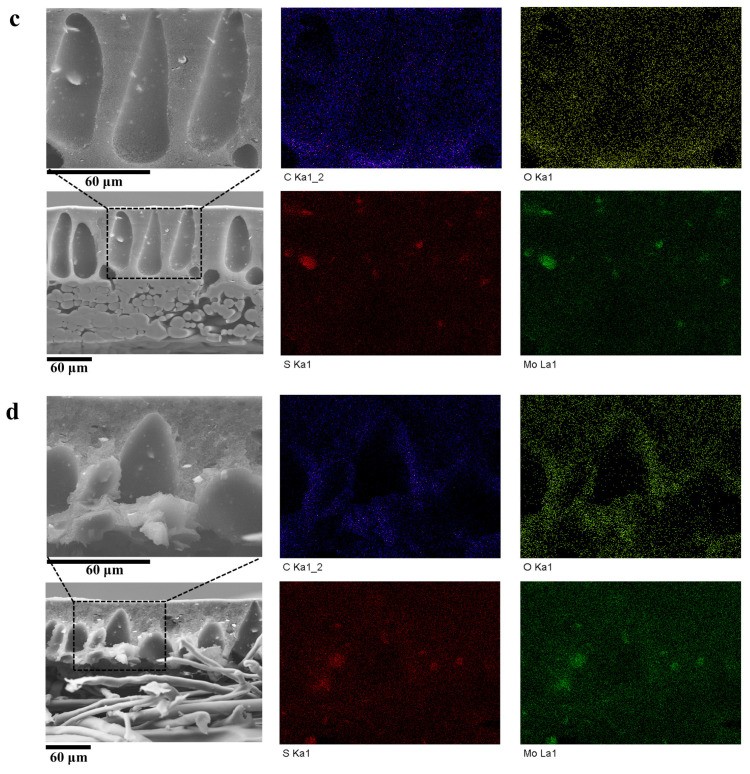
Elemental mappings of the cross-sections of the MF membrane filtered with industrial wastewater (**a**,**b**) and the UF membrane modified with MoS_2_ (**c**,**d**). The cross-sections were prepared using the frozen section technique (**a**,**c**) and cryogenically fractured with liquid nitrogen (**b**,**d**).

To further verify whether the cross-sections of the membranes prepared using the frozen section technique were suitable for the EDS elemental analysis, the cross-sections of the MF membranes filtered with industrial wastewater and the UF membranes modified with MoS_2_ were prepared using this method. The operating temperature of the cryotome was set at −30 °C, and the ultrapure water was used as the embedding medium. The fine cutting thicknesses were set to 0.5 µm. In Figure 13a, the PVDF layer of the MF membrane prepared using the frozen section technique mainly contained carbon and fluorine, and the deposit on the membrane surface contained mainly aluminum, iron, and oxygen. In contrast, the cross-section of the membrane cryogenically fractured with liquid nitrogen in Figure 13b was obviously uneven. The fibers were scattered and shielded some of the areas of the functional layer and the sediment. The sediment was dispersed due to the uneven force of the fracture. From Figure 13c, it could be seen that the PES layer of the UF membrane prepared using the frozen section technique contained mainly carbon and oxygen, and the MoS_2_ nanosheets of different sizes were distributed in it. The cross-section of the membrane in Figure 13d was cryogenically fractured with liquid nitrogen. Part of the functional layer was protruding, and it was difficult to distinguish whether some MoS_2_ materials were in situ from the morphology of the cross-section. On the one hand, the structural deformation or displacement of the membrane cross-sections directly led to the inaccurate elemental mapping analysis. On the other hand, an uneven sample surface could lead to the inaccurate analysis of the sample composition, as the X-ray detector we used was located on the side above the sample and the reception of the X-rays excited by the sample composition was influenced by the morphology [45]. The EDS results showed that the substances in the cross-section of the membrane prepared using the frozen section method were kept in the original state and could be accurately analyzed for composition and elemental distribution using EDS.

## 4. Economic Analysis

Table 1 lists the cost and the average processing time of cross-sectional sample preparation using the three different methods: liquid nitrogen cryogenic fracture, BIB polishing, and frozen section technique. Though the cost of the liquid nitrogen cryogenic fracture method is negligible, it produces the poorest quality in cross-sections. By comparing the instrument price, the cryostat microtome of the frozen section technique is much cheaper than that of the BIB polishing. Moreover, the cost of the sample preparation by the frozen section technique was 20 times lower than that of the BIB polishing, making it attractive for a wide range of applications. Moreover, the processing time of the frozen section technique is much shorter and can process multiple samples simultaneously. Overall, the frozen section technique proposed in this work could be promising in taking the place of the conventional liquid nitrogen cryogenic fracture and the expensive BIB polishing method.

## 5. Conclusions

In summary, a new frozen section technique was proposed to prepare cross-sections of membrane samples for SEM characterizations and EDS analysis. The processing of the membrane samples was assisted by a self-designed directional embedding mold and cut using a cryostat microtome. We found that the ultrapure water was a good embedding medium compared to the sucrose solutions. In addition, a lower working temperature of −30 °C and a fine cutting thickness of 0.5 µm were optimal in the preparation of the cross-sections. The MF, UF, and RO membranes all showed a very flat cross-sectional morphology without any damage as confirmed using the SEM characterizations. The pore structure, thickness, and the layered structure of the composite membranes were well characterized. Moreover, the industrial wastewater sediment on the cross-section of the MF membrane prepared using the frozen section technique showed accurate compositions using EDS analysis, and similarly, the MoS_2_ nanosheets modified on the nanocomposite UF membrane displayed clear elemental mappings. Compared to the conventional liquid nitrogen cryogenic fracture and the advanced BIB polishing, the frozen section technique was demonstrated to be efficient and to have an ease of operation. This method is not limited to polymeric membranes and can be applied to most solid materials. Due to its simplicity, high efficiency, low cost, and ease of implementation, this technique is practical for the preparation of large quantities of membrane samples.

## Figures and Tables

**Table 1:** The comparison of the instrument price, sample preparation price, and the sample processing time of the three methods.

Method	Instrument Price ($)	Sample Preparation Price ($)	Processing Time (h)
Liquid nitrogen cryogenic fracture	/	/	0.25
BIB polishing	120,000~290,000	140~220	3~5
Frozen section technique	25,000~38,000	7~15	1

## Data Availability

The data presented in this study are available on request from the corresponding author.
